# Multi-Parametric Electrochemical Sensing Platform: Applications in Animal Welfare

**DOI:** 10.3390/bios15050304

**Published:** 2025-05-10

**Authors:** C. Ferreira, E. Lynch, A. O’Herlihy, F. Barry, L. C. Nagle, S. R. Teixeira, P. Galvin

**Affiliations:** Tyndall National Institute, University College Cork, Lee Maltings, T12 R5CP Cork, Ireland; 120764015@umail.ucc.ie (E.L.); 120301093@umail.ucc.ie (A.O.); fiona-barry@hotmail.com (F.B.); lorraine.nagle@tyndall.ie (L.C.N.); sofia.teixeira@tyndall.ie (S.R.T.); paul.galvin@tyndall.ie (P.G.)

**Keywords:** sensors, dairy health, electrochemistry, electronics

## Abstract

The rapid growth of the dairy sector requires advanced monitoring tools to ensure sustainable practices that benefit the environment, economy, and human health. Current monitoring devices often lack multi-parametric capabilities, limiting their ability to provide comprehensive data on critical chemical and biochemical parameters. To address this challenge, this work presented the integration of a real-time multi-parametric device with sensors for pH, temperature, nitrate, and nitrite, providing a comprehensive solution to dairy cattle health monitoring. This solution included an electrochemical platform, Portable Unit for Lab-on-Site Electrochemistry (PULSE), and an application for data processing and display. In-house fabricated flexible gold-printed electrodes demonstrated accurate detection of nitrite and nitrate when integrated with the PULSE, achieving sensitivities of 6.32 μA/ppm/${\mathrm{cm}}^{2}$ in artificial interstitial fluid and 1.92 μA/ppm/${\mathrm{cm}}^{2}$ in phosphate buffered saline, respectively. The PULSE achieved 65.83% and 58.3% lower limits of detection in phosphate buffered saline than a benchtop potentiostat, for nitrate and nitrite, respectively, along with a 24.5% increase in nitrite sensitivity, enhancing its ability to detect lower analyte concentrations. pH sensing was carried out with a commercial screen-printed electrode coated with a layer of iridium oxide. The pH was tested in ruminal complex fluid, obtaining a pH sensitivity of −59.63 mV/pH and an accuracy of 98.9%. These findings highlighted the potential of this technology as an effective tool for dairy cattle health monitoring and its deployment in real-world scenarios.

## 1. Introduction

The dairy industry is a growing sector, with significant impacts on the environment, economy, human health, and diet-related issues [1,2]. The global dairy industry is projected to reach a valuation of USD 1243 billion by 2028 [3]. As the industry evolves, precise and comprehensive monitoring tools are increasingly necessary [1]. Real-time monitoring technologies play a critical role in this context by enabling the continuous assessment of key physiological and biochemical parameters in dairy cattle. Early and continuous monitoring allows for timely intervention, reducing disease incidence and antibiotic usage, thereby improving animal welfare and farm productivity and ultimately mitigating economic losses from disease outbreaks [4,5]. As demonstrated in recent studies, the integration of real-time data improves decision-making at the farm level, creating a positive feedback loop across animal health, environmental sustainability, and economic performance [6].

Research has resulted in health-monitoring devices for cattle, including wearable devices such as ear tags [7], leg braces [8], and collars [9,10]. However, these wearable devices primarily track physical activity and body temperature, offering limited insight into the animal’s internal biochemical status [11]. This lack of comprehensive physiological data constrains their utility in early disease detection and precision livestock management. Gathering this biochemical data could enhance advanced artificial intelligence (AI) models for farm management. Smart boluses have been developed to facilitate this process. These devices are inserted into the cow’s reticulum to collect valuable data [12,13,14]. Despite this advancement, most commercial smart boluses are limited to measuring only temperature and pH [15]. These parameters serve as vital indicators of dairy cattle health and productivity. In particular, subacute ruminal acidosis (SARA) is a prevalent disease affecting animal well-being that also poses a concern for human health [16]. SARA is characterized by a prolonged low ruminal pH, between 5.0 and 5.5, in contrast to the healthy range of 6.0 to 6.5 [17]. Internal body temperature readings remain unaffected by external factors, providing reliable indicators of fever and overall health [18]. Additionally, rumen temperature is correlated with various diseases, including mastitis [19], SARA [20], bovine respiratory disease (BRD) [21], and heat stress [22], and it has previously helped in detecting and quantifying water intake [23]. However, relying solely on temperature and pH restricts the diagnostic potential of these systems. This highlights the need for multi-parametric sensing platforms that can simultaneously monitor physical, chemical, and biochemical markers.

Cattle are frequently susceptible to nitrate poisoning, negatively impacting reproductive performance and milk production. Previous studies have reported that ingesting 0.22 g to 0.33 g of nitrate per kg of body weight can be fatal within three hours, corresponding to a nitrate concentration of 220 ppm to 330 ppm [24]. Hall [25] reported that poisoning occurred at nitrate levels of 300 ppm and nitrite levels of 20 ppm to 25 ppm in plasma or serum, while concentrations in ocular fluid were approximately two-thirds of these critical toxicity thresholds. Ruminants are especially vulnerable due to nitrate’s conversion to ammonia, with nitrite being an intermediate product during digestion [26]. During gestation, elevated nitrate levels reduce oxygen supply to the fetus, potentially causing abortion [27]. Nitrate also influence methane (${\mathrm{CH}}_{4}$) emissions, a significant greenhouse gas (GHG) [28]. Ruminant methane production from fermentation accounts for 90% of expelled methane from livestock [29]. In 2022, cattle were responsible for 62% of global livestock GHG emissions, with 54% attributed to ${\mathrm{CH}}_{4}$ from enteric fermentation [30]. Studies have explored using nitrate as a dietary supplement to mitigate ${\mathrm{CH}}_{4}$ emissions [24]. Therefore, monitoring ruminal nitrate and nitrite levels is vital for cattle health and supporting strategies to reduce GHG emissions. In addition, monitoring nitrate and nitrite levels in the rumen is essential for early disease detection. The accumulation of nitrite in the rumen can disrupt microbial activity, including inhibiting methanogens, which may reduce ${\mathrm{CH}}_{4}$ production but also impair feed digestibility. If nitrite is absorbed into the bloodstream, it can form methaemoglobin, decreasing the blood’s oxygen-carrying capacity and potentially leading to hypoxia, which can be fatal [31].

Typically, ruminal fluid sampling for nitrate and nitrite detection is obtained through oral stomach tubing or fistulated cows, with samples subsequently stored for later analysis [28]. However, this method faces challenges due to practical execution, animal comfort, saliva contamination, and potential sample quality compromise during storage. To overcome these issues and allow real-time detection, alternative techniques have proposed using electrochemical sensors [32]. A review by Lal et al. [33] highlighted the demand for robust sensors in multi-parametric scenarios, emphasizing repeatability, sensitivity, and selectivity. The literature has presented solutions that are mainly focused on water applications, requiring sampling. No identified study has simultaneously examined pH in the rumen while integrating other relevant parameters such as nitrate and nitrite, highlighting a gap in the literature that limits a comprehensive understanding of animal welfare. Developing a smart bolus prototype involved challenges such as durability in the rumen, measurement accuracy, and cost-effectiveness.

This study focused on integrating multiple key analytes, including pH, temperature, nitrate, and nitrite, into a single portable device called PULSE (Portable Unit for Lab-on-Site Electrochemistry) [34], enabling real-world biofluids measurements, such as with bovine ruminal fluid. By combining these sensing capabilities into one platform, this research aims to demonstrate the potential for continuous livestock health monitoring and early disease detection using an autonomous integrated sensor solution, such as the PULSE system. This is the first report of real-time nitrate and nitrite detection for bovine analysis using a portable and bolus-compatible system. This novel integration bridges a critical gap in current livestock-monitoring technologies by enabling the simultaneous tracking of both physical and chemical parameters in a single, deployable device.

## 2. Materials and Methods

### 2.1. Materials

Magnesium chloride (Mg${\mathrm{Cl}}_{2}$), sodium chloride (NaCl), potassium chloride (KCl), magnesium sulphate (MgS$O_{4}$), calcium chloride (Ca${\mathrm{Cl}}_{2}$), HEPES, mono-sodium phosphate (Na$H_{2}$P$O_{4}$), sucrose, sodium hydroxide (NaOH), nitric acid (HN$O_{3})$, potassium dicyanoargentate (KAg${\left(\mathrm{CN}\right)}_{2}$), potassium dicyanoaurate (KAu${\left(\mathrm{CN}\right)}_{2}$), lithium nitrate (LiN$O_{3}$), sodium nitrite (NaN$O_{2}$), iridium tetrachloride, and oxalic acid were obtained from Sigma Aldrich (Arklow, Ireland). Iridium (IV) chloride was purchased from Alfa-Aesar (Haverhill, MA, USA). Phosphate-buffered saline (PBS) solutions (10 mM) were obtained by dissolving 1 PBS tablet (pH 7.4) in 200mL of deionized (DI) water. Potassium hexacyanoferrate III and potassium hexacyanoferrate II solutions were prepared in PBS. All solutions were prepared with double distilled DI water with a resistivity of 18.2 MΩcm. Sterilized bovine rumen fluid was purchased from Bar Diamond, Inc. (Parma, ID, USA).

Artificial interstitial fluid (ISF) was prepared using an adaptation of the procedure provided in the literature [35]. A 200 mL artificial ISF solution was prepared by measuring out 0.7 mM of magnesium sulphate, 2.5 mM of calcium chloride, 1 mM HEPES, 3.5 mM potassium chloride, 123 mM sodium chloride, 1.5 mM mono-sodium phosphate, and 7.4 mM sucrose into 200mL of DI water. The final pH of the artificial ISF solution was 7.5. This composition was chosen to simulate a more complex biological environment than PBS, allowing for the better evaluation of the sensor’s performance.

Stock solutions of 200 ppm LiNO3 in 1 M NaOH and 200 ppm NaN$O_{2}$ in 10 mM PBS were made up for nitrate and nitrite analysis. The stock solutions were subsequently diluted to test various concentrations.

### 2.2. Equipment

All electrochemical measurements used the PULSE, except for the pH sensing repeatability test, which utilized a benchtop potentiostat to allow concurrent measurements. The PULSE was previously reported by Ferreira et al. [34], running in a tailored developed application (APP). For benchmarking and validating the results obtained by the PULSE, Methrom Multi Autolab MAC90389 (Metrohm, Runcorn, UK) was used with NOVA 2.1 Software. When using the benchtop instrument, the measurements were carried out at room temperature in a Faraday cage (Metrohm, UK). Carbon screen printed electrodes (SPEs) were purchased from DropSens (DRP-110GPH), consisting of a 4 mm diameter carbon working electrode (WE), carbon counter electrode (CE), and silver/silver chloride (Ag/AgCl) reference electrode (RE). A calibrated pH probe from Mettler Toledo (Greifensee, Switzerland) was used as a gold standard reference to ensure the accuracy of the measurements. A calibrated digital thermometer with a probe from Testo (Titisee-Neustadt, Germany) was used. An electrochemical sensor holder, accommodating a temperature sensor (NCP03 from Murata Electronics, Nagaokakyo, Japan) was 3D-printed, using a Formlabs Form 3 with standard white resin purchased from Formlabs (Somerville, MA, USA).

Preliminary results indicated that nitrate could not be detected using the commercial carbon SPEs or commercial gold sensors (C220AT from DropSens). Therefore, in-house gold-printed electrodes were fabricated to measure nitrate and nitrite concentrations. These electrodes offered a high degree of customization and the capacity for future integration into a smart bolus device. These electrodes were fabricated using a Voltera V1 printer (Voltera, Canada) [36], in polyimide (Kapton) films (50μm thickness). The WE was printed using gold polymer paste (C2041206P2 from Sun Chemicals, Alfreton, UK) with a 2 mm diameter, and silver paste (from Radionics RS, Dublin, Ireland) was added to improve the connection path. External Ag/AgCl RE and platinum wire CE were procured from IJ Cambria Scientific Ltd. (CH Instruments, Bee Cave, TX, USA).

### 2.3. Methods

#### 2.3.1. IrOx Functionalization

Prior to the analysis of pH, the SPEs were functionalized with an iridium oxide (IrOx) sensing layer. The IrOx functionalization process used was previously described in [34], and consisted of 80 Cyclic Voltammetry (CV) scans with a potential range from −0.7 V to 0.6 V at a scan rate of 10 mV/s.

#### 2.3.2. Flexible Gold Electrode Modification

**Figure 1:**
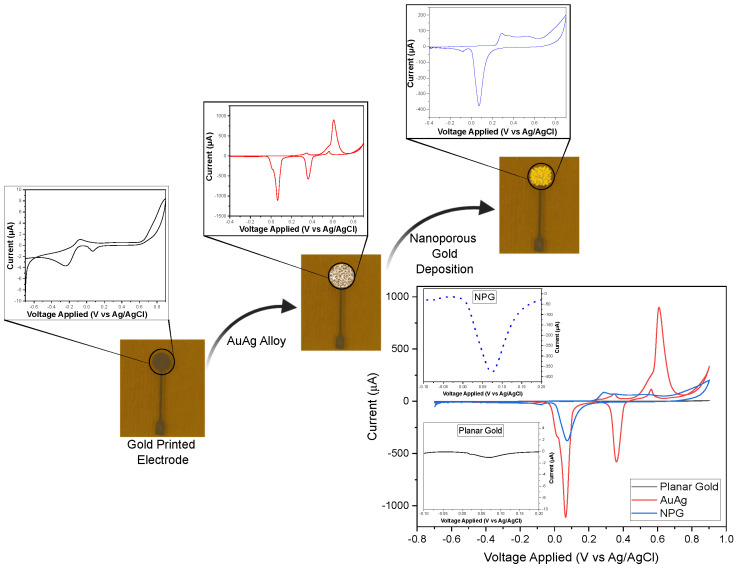
Schematic diagram of the NPG deposition process, where the potential was swept from −0.7 V to +0.9 V, with a scan rate of 10 mV/s in 1 M NaOH.

Previously to the analysis of nitrate and nitrite, unmodified gold-printed electrode surfaces of 2 mm in diameter were characterized using 1 M NaOH with CV, sweeping the potential from −0.7 V to 0.9 V at a scan rate of 10 mV/s. Following this, a nanoporous gold (NPG) deposition process, adapted from [37], was initiated due to its reported advantages in enhancing nitrite detection [38]. A gold–silver (AuAg) alloy was deposited onto the gold-printed electrode in a cyanide bath (20 mM $\mathrm{KAu}{\left(\mathrm{CN}\right)}_{2}$ and 100 mM $\mathrm{KAg}{\left(\mathrm{CN}\right)}_{2}$ in 250 mM ${\mathrm{Na}}_{2}{\mathrm{CO}}_{3}$) via chronoamperometry (CA) at −1.19 V for 180 s. The silver was then removed by submerging the electrode in 25% nitric acid for 30 min. A CV scan in 1 M NaOH was performed at each step for characterization, as illustrated in Figure 1.

#### 2.3.3. Electrode Characterization

Assessing the electrochemically active area of the electrodes is crucial for determining the sensitivity achieved during measurements. The Randles–Sevcik equation was employed to calculate the electrochemical active area of the WE in both the flexible gold-printed electrodes and the commercial SPE [39].

#### 2.3.4. pH Measurements

Open circuit potentiometry (OCP) measurements were performed at room temperature (≈20 °CC) on artificial ISF solutions with pHs varying from 3.0 to 11.0 (*n* = 2). These solutions were made using artificial ISF pH 7.5, and the pH was adjusted using hydrochloric acid and sodium hydroxide measured with a pH commercial probe, as applicable. The pH and temperature sensors were mounted on a 3D-printed holder to enable their immersion in a beaker containing the solution of interest (Appendix A).

To evaluate the system’s behaviour, measurements were taken in artificial ISF solutions for 200 s while the pH was gradually increased from 3.0 to 11.0 and then decreased back to 3.0 (*n* = 3). Additionally, to assess the stability over time of the IrOx deposition, pH measurements were performed using the benchtop potentiostat every 7 days for a total of 42 days (*n* = 3).

To evaluate the system’s response in the presence of other ions, interference tests were conducted on both the PULSE and the benchtop potentiostat. For this, 80 mM $\mathrm{Mg}{\mathrm{Cl}}_{2}$, 140 mM NaCl, and 30 mM KCl were separately dissolved in 10 mL DI water. These solutions were mixed separately with artificial ISF pH 3.0 solution using a 50/50 ratio. OCP measurements were carried out in triplicate on the four solutions.

#### 2.3.5. Nitrate and Nitrite Measurements

An analysis of nitrate was performed using CV with a potential window of 0 V to −1.3 V at 50 mV/s, while the nitrite sweep voltage ranged from 0 V to +0.9 V at 50 mV/s. Nitrate and nitrite analysis were carried out using NPG-modified electrodes with external Ag/AgCl reference and external platinum wire CE (Appendix A). For the validation of the measurements, the experiments were initially carried out in PBS buffer solutions at the PULSE and benchtop devices. Subsequently, nitrite analysis was exclusively carried out using the PULSE in artificial ISF to demonstrate its reliability for measurements in complex media.

To evaluate the device’s multi-parametric performance, nitrite, pH, and temperature were measured. Commercial SPEs modified with IrOx and NPG-modified electrodes were employed in the same beaker to enable the detection of pH and nitrites, respectively. To evaluate the influence of nitrite concentration on pH measurements, varying nitrite concentrations in ISF were tested while maintaining a fixed pH of 7.0. Additionally, to explore the reciprocal impact of pH on nitrite detection, a 100 ppm nitrite solution in ISF was examined across different pH levels.

The Limit of Detection (LOD)was determined from the calibration curves using the following Equation [40]:

(1) $$LOD=3.3\times \frac{SD}{m},$$

where *SD* represents the standard deviation of the blank samples, and *m* is the slope of the calibration curve. Additionally, the sensitivity was calculated as the ratio between the slope of the calibration curve and the electrochemical active area of the WE [41].

#### 2.3.6. Real-World Proof of Concept

The primary objective of this study is to demonstrate the suitability of the PULSE system for bovine health monitoring. To this end, this study evaluated the system’s capabilities for deployment in animal health monitoring by performing pH measurements in ruminal complex fluid across a range of 3.0 to 11.0.

## 3. Results and Discussion

### 3.1. Electrode Fabrication/ Modification

**Figure 2:**
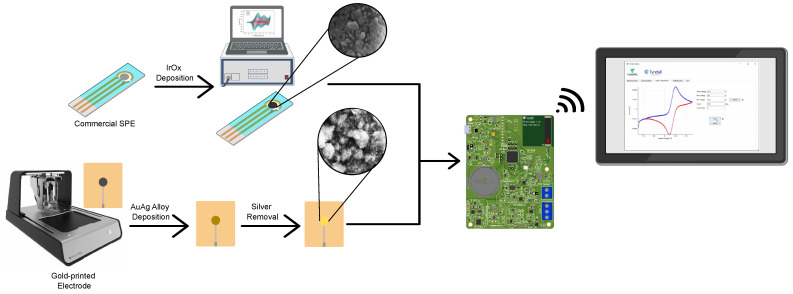
Schematic overview of the SPEs functionalized with IrOx, the in-house fabrication of gold-printed electrodes, and the silver removal for the creation of NPG layer, both applied to the PULSE device, with results displayed via the APP.

The commercial SPEs were effectively modified with an IrOx deposition layer to facilitate pH detection, resulting in an active area of $37.26\times 10^{-2}$ ${\mathrm{cm}}^{2}$. Additionally, in-house fabricated gold-printed electrodes were modified with NPG for nitrate and nitrite detection, achieving an active electrochemical area of 13.4 × ${\mathrm{10}}^{-2}$ ${\mathrm{cm}}^{2}$ after NPG deposition. These modified electrodes were integrated with the PULSE, which interfaces with smart devices, via a developed APP. This setup allows for real-time monitoring, as illustrated in Figure 2.

### 3.2. pH Measurements in ISF

**Figure 3:**
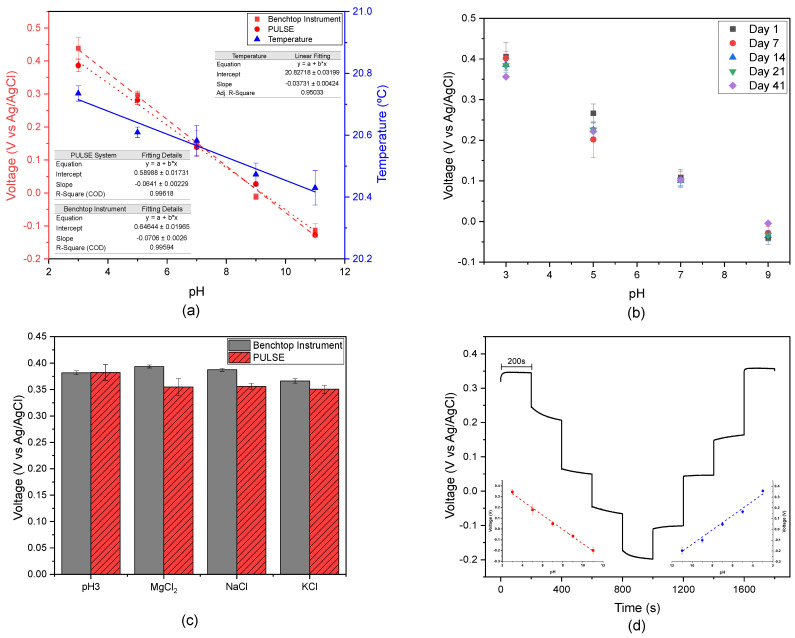
(**a**) Calibration plots for artificial ISF pH solutions across potential for both systems (*n* = 2, mean ± SD), along with the average temperature over a 200 s interval; (**b**) OCP measurements over 41 days (*n* = 3, except on day 41 where *n* = 2, mean ± SD); (**c**) interference results on an ISF pH 3.0 solution in the presence of ${\mathrm{MgCl}}_{2}$, NaCl, and KCl, using both systems (*n* = 3, mean ± SD); and (**d**) OCP responses of pH sensors to different pH solutions in ISF, increasing and decreasing the pH levels, and corresponding calibration curves obtained using the PULSE system (*n* = 3, mean ± SD).

OCP measurements were conducted on artificial ISF with various pH values. The purpose was to investigate the pH behaviour of complex solutions and benchmark the PULSE against the benchtop instrument. Simultaneously, temperature measurements were taken using a thermistor connected to the PULSE system. In Figure 3a, the calibration curves obtained from both systems are illustrated, along with the corresponding temperature measurements. The regression analysis conducted with the PULSE system revealed a slope of −64.1 mV/pH with an $R^{2}$ value of 0.996, which is close to the ideal −59 mV/pH, according to the Nernst Equation [42]. Consequently, the accuracy of the measurement performed by the PULSE was 91.4%. Similarly, for the benchtop potentiostat, the regression analysis yielded a Nernst response with a slope of −70.6 mV/pH and an $R^{2}$ value of 0.996. The results showed a strong correlation (>0.99) between the datasets from the benchtop instrument and the PULSE, highlighting the PULSE’s potential as an alternative for pH sensing in complex media. Furthermore, the temperature measurements exhibited a linear response as described in the literature [43], showing a slight decrease as the pH increased.

#### 3.2.1. Repeatability

A repeatability study was conducted in the benchtop potentiostat, involving testing artificial ISF pH solutions over a period of 41 days at intervals of 7 days, as illustrated in Figure 3b. The values obtained at pH 7.0 exhibited remarkable stability throughout the entire duration of the study. In contrast, pH 5.0 readings displayed greater variability from day 1 to day 7; however, subsequent days converged towards day 7. This difference can be attributed to the IrOx stabilization time. Therefore, in the future, IrOx deposition should be performed 7 days after preparing the solution. High correlations (>0.9) were consistently observed between the datasets across multiple days, supporting the sensors’ suitability for extended use, due to their precision and accuracy.

#### 3.2.2. Interferences

An interference study was performed and the results obtained are depicted in Figure 3c. The voltage differences measured between the artificial ISF solution at pH 3.0 and various interference solutions are summarized in Table 1, highlighting the performance of the benchtop instrument and the PULSE device when exposed to solutions of ${\mathrm{MgCl}}_{2}$, NaCl, and KCl. The highest voltage difference using the benchtop instrument was observed with KCl (0.0156 V), indicating an interaction with the main solution [44]. Similarly, the PULSE system exhibited its largest voltage difference with KCL (0.0316 V, representing an 8.3% interference). The behaviour of the systems in the presence of KCl suggested that potassium ions, known to increase solution basicity, contributed to a decrease in measured potential, consistent with findings reported in previous studies [45]. Similarly, magnesium ions have alkalinizing properties [46], as evidenced in the PULSE. In contrast, the benchtop instrument showed a different response pattern, which could be due to its complexity and advanced signal processing. Despite these differences, both systems accurately measured the pH of the artificial ISF solution at pH 3.0 in the presence of interfering ions, with percentage errors below 10%, within the acceptable range reported in the literature [47,48].

#### 3.2.3. pH Staircase

To evaluate the system performance in a dynamic environment, artificial ISF solutions with different pH levels were prepared. OCP measurements were conducted for 200 s in each solution, with the pH values incrementally increasing from 3.0 to 11.0 and then decreasing back to 3.0. Figure 3d illustrates the distinct voltage changes observed each time the solution’s pH was altered, giving it a stair shape. In some instances, the readings required a brief period to stabilize after the change, due to the sensor’s behaviour. Calibration curves were constructed for both the increasing and decreasing pH scenarios, resulting in slopes of −66.56 mV/pH and 68.72 mV/pH, respectively. In both situations, the calibration curves are consistent with Nernstian behaviour [42]. These findings suggested that the measurement system can accurately measure pH.

### 3.3. Nitrate Detection

To evaluate the detection of nitrate, solutions with nitrate concentrations ranging from control (10 mM PBS and 0 ppm nitrate) to 200 ppm of nitrate in 10 mM PBS solution were assessed. NPG-modified gold-printed electrodes were used to enhance nitrate detection. This modification increased the electrochemical active area of the WE, thereby improving detection (Appendix B). For both the benchtop instrument and the PULSE, NPG-modified printed electrodes (*n* = 2) were used. CV scans were taken with the voltage ranging from 0 V to −1.3 V at 50 mV/s, where the typical reduction reaction of nitrate ($NO_{3}^{-}$) to nitrite ($NO_{2}^{-}$) occurred as follows:

(2) $$NO_{3}^{-}+2H^{+}+2E^{-}\to NO_{2}^{-}+H_{2}O$$

**Figure 4:**
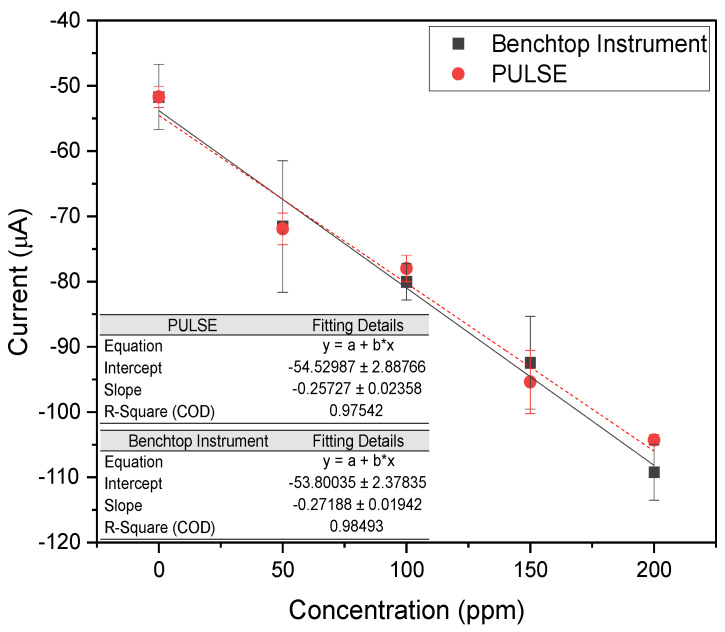
Calibration plots of peak reduction currents across multiple concentrations of nitrate in PBS, using the benchtop instrument, and the PULSE System (*n* = 2, mean ± SD).

The reduction potential analyzed ranged between −0.8 V and −1.1 V, looking at the lowest measured current, representing the peak reduction current. A calibration plot was made comparing the measured current against the nitrate concentration, as shown in Figure 4.

The benchtop instrument and the PULSE system exhibited high linearity, with $R^{2}$ values of 0.985 and 0.975, respectively. The results revealed a high correlation (>0.99) between the datasets of the two systems, supporting that the PULSE system can accurately measure nitrate with a LOD of 20.75 ppm (0.335 mM) and a sensitivity of 1.92 $\mu A/\mathrm{ppm}/{\mathrm{cm}}^{2}$ (0.119 $\mathrm{mA}/\mathrm{mM}/{\mathrm{cm}}^{2}$). The benchtop instrument yielded a LOD of 60.73 ppm (0.979 mM) and a sensitivity of 2.03 $\mu A/\mathrm{ppm}/{\mathrm{cm}}^{2}$ (0.122 $\mathrm{mA}/\mathrm{mM}/{\mathrm{cm}}^{2}$). The PULSE achieved a 65.83% lower LOD than the benchtop instrument, meaning the PULSE can detect smaller concentrations of nitrate. The slightly higher (2.46% in mM) sensitivity of the benchtop instrument suggested it generated a stronger signal per unit concentration. Nevertheless, the PULSE offered better detection limits with comparable sensitivity, reinforcing its suitability for real-world applications.

### 3.4. Nitrite Detection

CV measurements were conducted with a sweeping voltage ranging from 0 V to +0.9 V at 50 mV/s. Typically, nitrite oxidation occurs at approximately 0.77 V [38], with the following reaction:

(3) $$NO_{2}^{-}+H_{2}O\to NO_{3}^{-}+2H^{+}+2e^{-}$$

Thus, the potential analysed was between 0.5 V and 0.9 V, with the maximum current measured within this range.

**Figure 5:**
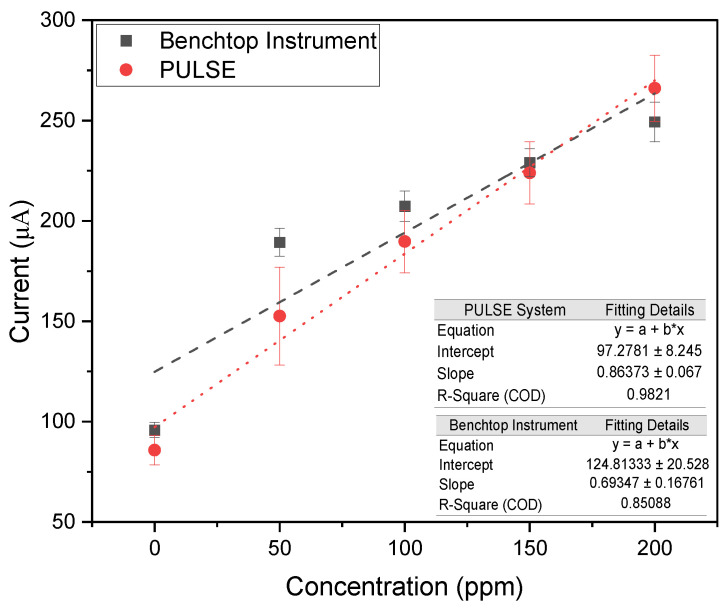
Calibration plots of peak oxidation currents for varying nitrite concentrations in PBS measured with the benchtop instrument and the PULSE system (*n* = 3, mean ± SD).

An initial analysis of nitrite was conducted in PBS using the benchtop instrument and the PULSE. Nitrite concentrations were varied, starting with a control solution of 10 mM PBS containing 0 ppm nitrite and increasing up to 200 ppm nitrite. A calibration plot was generated, comparing the measured current against the nitrite concentration, as shown in Figure 5.

The benchtop instrument and the PULSE exhibited high linearity, with $R^{2}$ values of 0.85 and 0.98, respectively. The datasets from both systems were closely aligned, and an additional correlation analysis indicated a strong correlation (>0.9) between the systems. These results confirmed that the PULSE system can accurately measure nitrite concentrations. The PULSE yielded a LOD of 14.62 ppm (0.318 mM) and a sensitivity of 6.45 $\mu A/\mathrm{ppm}/{\mathrm{cm}}^{2}$ (0.297 $\mathrm{mA}/\mathrm{mM}/{\mathrm{cm}}^{2}$). In comparison, the benchtop instrument demonstrated a LOD of 35.08 ppm (0.762 mM) and a sensitivity of 5.18 μA/ppm/${\mathrm{cm}}^{2}$ (0.238 $\mathrm{mA}/\mathrm{mM}/{\mathrm{cm}}^{2}$). Similarly to nitrate detection, the PULSE achieved a lower LOD, representing a 58.3% improvement. This means that the PULSE can detect significantly lower concentrations of the target analyte. The PULSE exhibited a 24.5% higher sensitivity than the benchtop instrument, meaning a better signal response per unit concentration. The significantly lower LOD and higher sensitivity reinforce the PULSE’s practical utility for real-world deployment.

**Figure 6:**
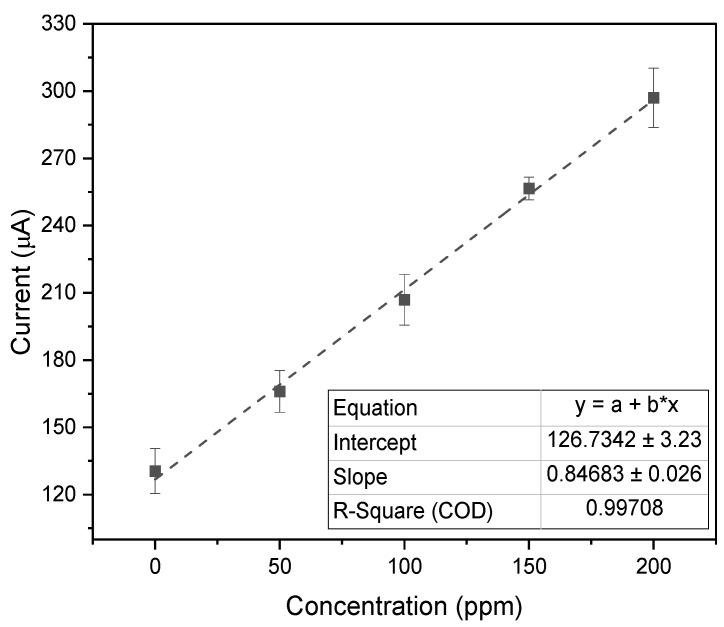
Calibration plot of peak oxidation currents across multiple concentrations of nitrite in ISF, using the PULSE System (*n* = 3, mean ± SD).

Subsequent analyses were performed in artificial ISF. Nitrite concentrations varied from the control (artificial ISF and 0 ppm of nitrite) to 200 ppm of nitrite. A calibration plot compared the measured current against the nitrite concentration, as depicted in Figure 6. The system exhibited high linearity, with $R^{2}$ of 0.997, a LOD of 39.27 ppm (0.854 mM), and a sensitivity of 6.32 $\mu A/\mathrm{ppm}/{\mathrm{cm}}^{2}$ (0.291 $\mathrm{mA}/\mathrm{mM}/{\mathrm{cm}}^{2}$).

**Figure 7:**
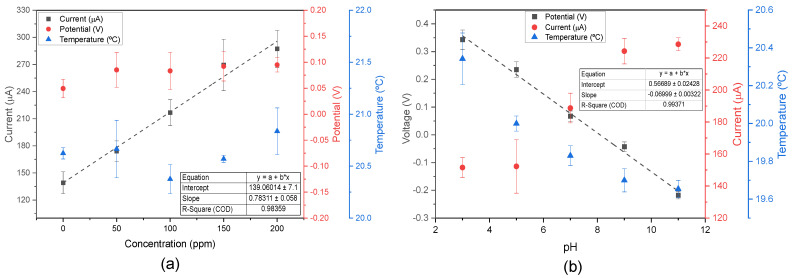
(**a**) Calibration plot of peak oxidation currents across multiple concentrations of nitrite at pH 7.0 (*n* = 3, mean ± SD); and (**b**) pH calibration plot in artificial ISF and 100 ppm nitrite solution (*n* = 3, mean ± SD), and average current detecting 100 ppm nitrite across different pH levels.

Furthermore, an examination of Figure 7a revealed that different nitrite levels could be measured at pH 7.0. According to the pH calibration curve in the ISF staircase (Figure 3d), the measured voltage at pH 7.0 was approximately 0.0499 V. In the control solution (only artificial ISF), a voltage of 0.0494 V was measured, matching the previous results. In the presence of nitrite, the voltage increased slightly to ≈0.088 V, and remained stable across different nitrite concentrations, indicating that nitrite presence does not significantly interfere with pH detection. Additionally, temperature measurements were minimally affected by external factors.

In contrast, Figure 7b illustrated the behaviour of a 100 ppm nitrite solution across varying pH levels. According to the nitrite calibration curve in artificial ISF at pH 7.5 (Figure 7a), the measured current at 100 ppm was 206.97 μA. At pH 7.0, the measured current was 188.72 μA, representing a small difference of 18.25 μA. This discrepancy can be attributed to variations in pH and sensors. Even in more acidic solutions, the nitrite peak oxidation remained detectable, despite being reduced. Nitrite peak oxidation exhibits pH-dependent behaviour, decreasing in acidic conditions and increasing in basic environments, as previously reported in the literature [49]. In the future, more in-depth studies on nitrite and pH should be conducted to create a matrix that uses different nitrite calibration curves based on pH levels. The pH detection remained unaffected, and a calibration curve with a slope of −69.9 mV/pH was established, following a Nernst behaviour [42]. Additionally, the literature supported the observation that temperature decreases with increasing pH [43].

### 3.5. Real World Proof of Concept

**Figure 8:**
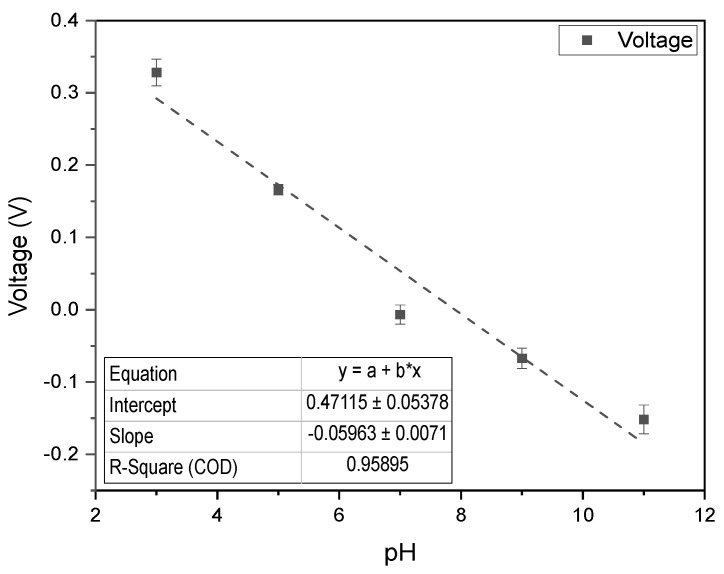
pH calibration curve in ruminal fluid ranging from 3.0 to 11.0, using the PULSE system (*n* = 3, mean ± SD).

To evaluate the PULSE system for deployment in animal health monitoring, pH measurements were taken in ruminal complex fluid. The resulting calibration curve, shown in Figure 8, exhibited an $R^{2}$ value of approximately 0.96 with a slope of −59.63 mV/pH, consistent with Nernstian behaviour [42], and an accuracy of 98.9%. These findings emphasized the accuracy and utility of the PULSE system and provided strong support for its practical application in real-world scenarios.

## 4. Conclusions

This study established a major advancement in livestock health monitoring by demonstrating the successful integration of pH, temperature, nitrate, and nitrite sensors into the PULSE platform. This work is the first to report the real-time detection of both nitrate and nitrite in the context of bovine analysis using a portable sensing system, filling a critical gap in the literature where no prior studies have combined the measurement of these biochemical markers with physical and chemical parameters in a smart bolus-compatible format.

The PULSE has enabled accurate measurements of pH, nitrite, and nitrate in PBS and artificial ISF. pH detection in ISF demonstrated excellent linearity ($R^{2}>0.99$), with a sensitivity of −64.1 mV/pH and 91.4% accuracy. Additionally, this system demonstrated capability for nitrite and nitrate detection, with sensitivities of 6.32 μA/ppm/${\mathrm{cm}}^{2}$ in artificial ISF and 1.92 μA/ppm/${\mathrm{cm}}^{2}$ in PBS, respectively. Furthermore, compared to the benchtop instrument, the PULSE achieved a 65.83% lower LOD for nitrate in PBS solution. Similarly, for nitrite measurement in PBS, the PULSE exhibited a 58.3% lower LOD and a 24.5% increase in sensitivity, demonstrating its improved capability for detecting lower analyte concentrations. The pH detection in rumen fluid demonstrated an accuracy of 98.9%, highlighting the PULSE system’s potential for advancing sustainable practices in the dairy sector, with potential impacts on economic efficiency and human health. These results highlighted the PULSE’s capability to accurately measure nitrate and nitrite, critical indicators of animal health with an indirect relationship with GHG emissions, while demonstrating its practicality through testing in complex ruminal fluid for real-world bovine health monitoring.

The findings of this study lay the groundwork for future research exploring multi-modal sensing in precision agriculture, with PULSE positioned as a pioneering platform in this emerging field. Before the in vivo deployment of the smart bolus technology, several critical steps must be completed. These include mechanical testing for structural integrity, pressure and temperature tolerance, and the development of robust sealing to prevent fluid ingress. The long-term durability and biofouling resistance of the sensors must be evaluated, along with cleaning strategies. Power consumption needs to be optimised to support extended autonomous operation, and wireless communication must be validated in biological environments. Future work will also focus on the integration of PULSE into smart bolus form factors, as well as its connection to Internet of Things (IoT) networks for continuous remote monitoring and data-driven decision-making. Finally, regulatory, ethical, and animal welfare aspects must be considered for future use on animals.

## Figures and Tables

**Table 1:** Voltage difference (V) between the artificial ISF pH 3.0 solution and the interference solutions (${\mathrm{MgCl}}_{2}$, NaCl, and KCl) for the benchtop instrument and the PULSE.

System	${\mathrm{MgCl}}_{2}$	NaCl	KCl
Benchtop Inst.	0.0119 V	0.0058 V	0.0156 V
PULSE	0.0274 V	0.0265 V	0.0316 V

**Figure A1:**
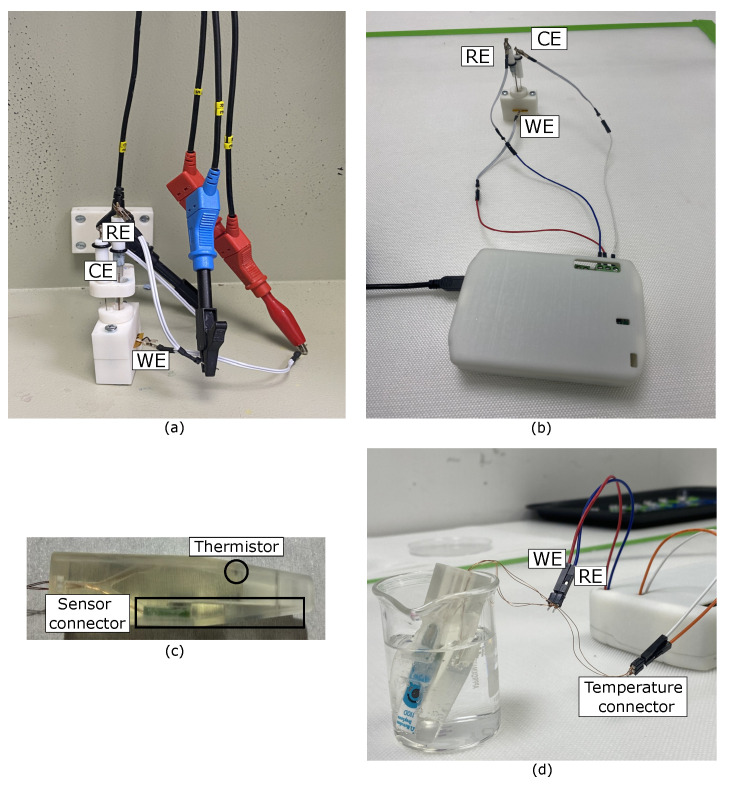
(**a**) Electrochemical setup inside the Faraday cage, featuring the 3D-printed holder designed to facilitate electrochemical measurements; (**b**) setup for measurements using the PULSE; (**c**) 3D-printed holder for immersive in-liquid measurements; and (**d**) setup for measurements using the PULSE for pH measurements.

**Figure A2:**
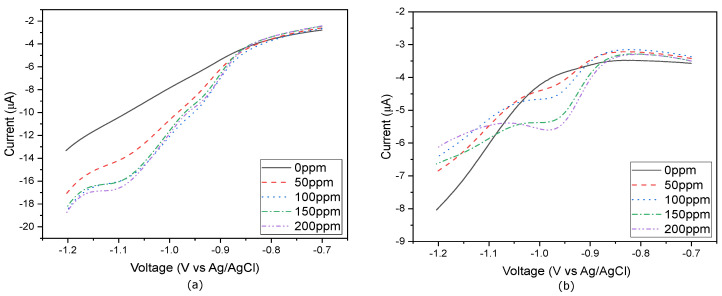
CV scans detecting nitrate from 0 ppm to 200 ppm in PBS, conducted (**a**) using a control gold flexible electrode, and (**b**) after NPG deposition.

## Data Availability

The original contributions presented in this study are included in the article. Further inquiries can be directed to the corresponding author.

## Abbreviations

The following abbreviations are used in this manuscript:

AI	artificial intelligence
APP	application
BRD	bovine respiratory disease
CA	chronoamperometry
CE	counter electrode
CV	cyclic voltammetry
DI	deionized
GHG	greenhouse gas
IrOx	iridium oxide
ISF	interstitial fluid
LOD	limit of detection
NPG	nanoporous gold
OCP	open circuit potentiometry
PBS	phosphate buffered saline
PULSE	portable unit for lab-on-site electrochemistry
RE	reference electrode
SARA	subacute ruminal acidosis
SD	standard deviation
SEM	scanning electron microscope
SPE	screen-printed electrode
WE	working electrode
